# Therapeutic Targeting of Nonalcoholic Fatty Liver Disease by Downregulating SREBP-1C Expression *via* AMPK-KLF10 Axis

**DOI:** 10.3389/fmolb.2021.751938

**Published:** 2021-11-12

**Authors:** Yu-Chi Chen, Rong-Jane Chen, Szu-Yuan Peng, Winston C. Y. Yu, Vincent Hung-Shu Chang

**Affiliations:** ^1^ Department of Biotechnology, National Kaohsiung Normal University, Kaohsiung, Taiwan; ^2^ Department of Food Safety/Hygiene and Risk Management, College of Medicine, National Cheng Kung University, Tainan, Taiwan; ^3^ School of Medical Laboratory Science and Biotechnology, Taipei Medical University, Taipei, Taiwan; ^4^ The PhD Program for Translational Medicine, College of Medical Science and Technology, Taipei Medical University, Taipei, Taiwan; ^5^ Germmine Bio, Yongkang Dist., Tainan, Taiwan

**Keywords:** Krüppel-like factor 10, nonalcoholic fatty liver disease, AMP-activated protein kinase, sterol regulatory element-binding Protein-1, lipogenesis

## Abstract

Krüppel-like factor 10 (KLF10) is a phospho-regulated transcriptional factor involved in many biological processes including lipogenesis; however, the transcriptional regulation on lipogenesis by KLF10 remains largely unclear. Lipogenesis is important in the development of nonalcoholic fatty liver disease (NAFLD) which was known regulated mainly by AMP-activated protein kinase (AMPK) and sterol regulatory element-binding protein (SREBP-1C). Interesting, our previous study using phosphorylated site prediction suggested a regulation of AMPK on KLF10. Therefore, we aimed to study the protein–protein interactions of AMPK on the regulation of KLF10, and to delineate the mechanisms of phosphorylated KLF10 in the regulation of NAFLD through SREBP-1C. We performed *in vitro* and *in vivo* assays that identified AMPK phosphorylates KLF10 at Thr189 and subsequently modulates the steady state level of KLF10. Meanwhile, a chromatin immunoprecipitation–chip assay revealed the novel target genes and signaling cascades of corresponding to phosphorylated KLF10. SREBP-1C was identified as a target gene suppressed by phosphorylated KLF10 through promoter binding. We further performed high-fat-diet-induced NAFLD models using hepatic-specific KLF10 knockout mice and wild-type mice and revealed that KLF10 knockout markedly led to more severe NAFLD than that in wild-type mice. Taken together, our findings revealed for the first time that AMPK activates and stabilizes the KLF10 protein *via* phosphorylation at Thr189, thereby repressing the expression of SREBP-1C and subsequent lipogenesis pathways along with metabolic disorders. We suggested that the targeted manipulation of liver metabolism, particularly through increased KLF10 expression, is a potential alternative solution for treating NAFLD.

## Introduction

Nonalcoholic fatty liver disease (NAFLD) is characterized by the accumulation of fat in the liver that can lead to a more advanced stages of nonalcoholic steatohepatitis (NASH), including cirrhosis, end-stage liver disease, and hepatocellular carcinoma (Friedman et al., 2018). NAFLD is associated with obesity, insulin resistance, dyslipidemia, cardiovascular disease and malignancy (Lindenmeyer and McCullough, 2018). The global prevalence of NAFLD is estimated at ∼25% and has been recognized as an increasing worldwide health issue. However, adequate therapy for NAFLD and NASH is still lacking. Accordingly, understanding the key mechanisms of NAFLD could help with the development of diagnostic and therapeutic strategies to mitigate its global impact. De novo lipogenesis is one of the major mechanisms contributing to increased fatty acid (FA) transportation and accumulation in the liver (Moon, 2017). This pathway is mainly regulated by transcription factors including sterol regulatory element binding protein-1C (SREBP-1C), carbohydrate-responsive element-binding protein (ChREBP), liver X receptor α (LXR-α), and peroxisome proliferator-activated receptor γ (PPAR-γ) (Moon, 2017). SREBP-1C regulates the expression of genes that catalyze the synthesis of FAs, triglycerides (TGs), and NADPH required for FA synthesis (Moon, 2017). In addition, AMP-activated protein kinase (AMPK) is known as a crucial regulator of energy metabolism in the liver, adipose tissue, and muscle, and is particularly closely associated with hepatic lipogenesis and FA oxidation. AMPK regulates lipogenesis through the activity of enzymes including acetyl-CoA carboxylase (ACC), SREBP-1, and fatty acid synthase (FAS), *via* phosphorylation and dephosphorylation (Kim et al., 2020). The substrates of AMPK such as Insig and Sirt 1 was also reported to modulate SREBP-1C activity (Han et al., 2019; Chyau et al., 2020). This indicated that AMPK could regulate SREBP-1C through additional mediators. Interesting, we recently reported that Krüppel-like factor 10 (KLF10) expression is upregulated upon activation of AMPK (Chang et al., 2017), and KLF10 could suppress lipogenic gene expression (Iizuka et al., 2011). In this regard, we proposed the potential mechanism of AMPK-KLF10 axis in regulating NAFLD *via* downregulation of SREBP-1C.

KLF10 also called TGF-inducible early gene 1 (TIEG1) which is a transcription factor belonging to the Krüppel-like family that is characterized by a DNA-binding domain that contains 3 C2H2-type zinc fingers capable of binding to a CACCC-element or GC-box in the promoter region of target genes, thereby regulating gene expression (Lee et al., 2020). KLF10 also competitively binds to GC-rich, Sp1-like sequences of target promoters to modulated multiple physiological and pathological processes, including glucose and lipid metabolism, bone homeostasis, cardiac hypertrophy, neovascularization, cell growth, and tumorigenesis as a negative or positive regulators (Subramaniam et al., 2010; Hsu et al., 2011; Wu et al., 2015; Pollak et al., 2018; Lee et al., 2020). Notably, KLF10 was reported to affect hepatic and systemic metabolism, and KLF10 acts as a gluconeogenesis inhibitor by inhibiting the phosphoenolpyruvate carboxykinase (Pepck) gene, which is associated with hepatic glucose output (Guillaumond et al., 2010). The Klf10^−/−^ mouse presented downregulated expression of glycolytic proteins, upregulated expression of gluconeogenic and lipogenic proteins, and elevated blood TG levels in female mice (Guillaumond et al., 2010). KLF10 is further capable of inhibiting the expression of ChREBP and its target genes in rat hepatocytes; therefore, a negative feedback loop exists between KLF10 and gluconeogenesis (Iizuka et al., 2011). In addition, KLF10 expression in primary hepatocytes was increased in response to PPARα activation, which promotes FA oxidation, indicating that KLF10 has a protective role in hepatic steatosis (Rakhshandehroo and Hooiveld, 2009; Iizuka et al., 2011). However, the role and regulatory mechanisms of KLF10 in hepatic lipogenesis remains unclear that led us to further investigate its protective role in hepatic lipogenesis through SREBP-1C and its related pathways.

The regulation of KLF10 protein activity is complex, and the detailed mechanisms remain largely unclear. KLF10 is likely to be post-translationally modified, which makes it more stable or easy degradation. Phosphorylation appears to be a common posttranslational modification of KLF10 and seems to be a mechanism that regulates its transcriptional activity. Our previous studies indicated that Raf-1 phosphorylated KLF10 leading to its destabilization by binding to peptidyl-prolyl cis–trans isomerase NIMA-interacting 1 (PIN1), thus decreasing the ability of KLF10 to inhibit tumor progression (Hwang et al., 2013). We have also indicated that cyclin-dependent kinase 2 (CDK2) upregulates the protein level of KLF10 and phosphorylates KLF10 by reducing its association with SIAH1, a KLF10 E3-ubiquitin ligase (Lin et al., 2015). Notably, KLF10 is a critical transcription factor that downregulates lipogenesis, but the mechanisms remained unclear (Hsu et al., 2011). At present, the effective preventive and therapeutic agents targeted to the pathways regulating NAFLD are still lacking. Therefore, we sought to identify whether KLF10 could be a novel and potential target to prevent NAFLD. Our study is the first one to provide the solid evidence that AMPK is an upstream kinase that phosphorylates KLF10 at Thr189, resulting in the protein interaction and the repressive activity on SREBP-1C leading to downregulation of NAFLD. The present results have also provided a novel insight into how KLF10 prevents NAFLD, which could be a preventive target for NAFLD.

## Materials and Methods

### Ethics Statement and Animal Study

This is a simple comparative study for investigating the role of KLF10 in the preventive effect for NAFLD. All animal experiments were conducted in accordance with the guidelines established by the Laboratory Animal Center of Taipei Medical University (TMU), Taiwan. The animal use protocols were reviewed and approved by the TMU Institutional Animal Care and Use Committee. KLF10-deficient mice were created as described in our previous publication (Huang et al., 2016). In brief, the KLF10 gene targeted embryonic stem cells (B6129) were induced deletion by Cre recombinase and then used to generate chimeric mice (C57BL/6J blastocyst). Chimeric males were used as founders to generate homozygous KLF10^−/−^, and genomic DNA was isolated from tail biopsies of agouti-colored offspring and screened for germ line transmission of the null allele by PCR and Southern blot (primers and probes have been shown in previous publication (Huang et al., 2016)). Heterozygous male KLF10 mutant mice were bred to C57BL/6 females to increase the colony size, and subsequent heterozygous male and female mice were interbred to generate homozygotes. All homozygotes used in our experiments had a mixed genetic background between B6129 and C57BL/6 over 15 generations. The 6 wild type (WT) C57BL/6J mice and 6 KLF10 KO C57BL/6J male mice (6 weeks old) were randomized divided into two groups and were fed either a high-fat diet (HFD, TestDiet 58Y1; 60% kcal provided by fat) or a normal diet (LabDiet-5001; containing 5% fat) for up to 8 weeks. This is not a blinded study because the objective is to investigate KLF10 gene function in the regulation of NAFLD. No animals were withdrawal during the study. After sacrificed, livers were collected for further immunohistochemistry and oxygen consumption rate (OCR) analysis.

### Western Blotting and Co-immunoprecipitation (Co-IP)

HepG2 human liver hepatocellular cells (ATCC^®^ HB-8065^™^) were cultured in DMEM (Sigma-Aldrich, Louis, MO, United States) supplemented with 10% fetal bovine serum (FBS, Biological Industries, Cromwell, CT, United States) at 37°C in 5% CO_2_. After treatment, proteins were extracted for Western blotting assay according our previous studies (Huang et al., 2016). For Co-IP, cell extracts were incubated with antibodies for 1 h at 4°C with rotation, followed by incubation with protein G Sepharose (Merck Millipore, Burlington, MA, United States) for 4 h then collected by centrifugation and washed. The bound proteins were analyzed by Western blotting using antibodies as indicated.

### GST (Glutathione S-Transferase) Fusion Proteins and Pull-Down Assay

GST-tagged KLF10 plasmids were transformed into *E. coli* BL21-CodonPlus^®^ (DE3)-RIPL (Agilent, Santa Clara, CA, United States) and purified according to standard protocols. For the pull-down assays, equal amounts of GST and GST fusion proteins were incubated with cellular proteins. The bound proteins were directly released from the beads using sodium dodecyl sulfate buffer and analyzed by Western blotting.

### *In vitro* Kinase Assay

For the *in vitro* kinase assay, GST-fusion proteins were used as substrates. The substrates were combined with recombinant AMPKs in reaction buffer and incubated at 30°C for 30 min. The labelled proteins were then resolved by SDS-PAGE and visualized by autoradiography.

### Protein Stability Measurement

For the protein stability assays, H1299 cells were transfected using Lipo 2000 (Invitrogen, Carlsbad, CA, United States) with plasmid containing HA-KLF10 Thr189A or Thr189D mutants to further study the mechanisms of AMPK on the regulation of KLF10. Cells were treated with cycloheximide (Sigma-Aldrich, Louis, MO, United States) at 24 h post-transfection. Cell extracts were prepared as described above, followed by immunoblotting.

### Chromatin Immunoprecipitation-Chip (ChIP-Chip) Assay

Total genomic DNA was isolated and sonicated using the EZ-Chip™ Kit (Merck Millipore: Burlington, MA, United States) according to the protocol. Each ChIP fragment was linked, amplified and printed onto human promoter arrays (Roche NimbleGen Inc.: Pleasanton, CA, United States). These arrays were probed with Cy3-labeled DNA from the anti-KLF10 immunoprecipitants and Cy5-labeled background DNA (anti-IgG). The three independent KLF10 ChIP-chip results were aligned to observe possible candidates for pathway prediction (Hsu et al., 2011). One candidate, the SREBP-1C, was selected and then characterized in this study.

### Plasmid Construction and Promoter Luciferase Assay

DNA fragments of interest, covering nearly 3.3 kb upstream of the mouse *SREBP-1C* promoter region, were separately cloned into the pGL3-Enhancer vector (Promega Corporation, Madison, WI, United States). Before they were cloned, each fragment was amplified using PCR with specific primers (forward: 5′-CCg Cgg ggg Cgg ggC Tgg TC-3′; reverse: 5′-ggC gCA gCC TCC gg-3′). Luciferase activity was determined using a Dual-Luciferase Reporter Assay (Promega Corporation, Madison, WI, United States) after transfection.

### mRNA Expression Analysis

Total RNA was amplified by a Low Input Quick-Amp Labeling kit (Agilent Technologies, Santa Clara, CA, United States) and labeled with Cy3 (CyDye, Agilent Technologies, Santa Clara, CA, United States) during the *in vitro* transcription process. The mRNA expression profiles were obtained using the Affymetrix mouse 430 2.0 array. Raw data were imported into ArrayTrack v 3.1.5 for further analysis. Microarray data were normalized by the MAS 5.0 algorithm and were further normalized per chip to the same median intensity value of 1,000. The Welch *t*-test within ArrayTrack^®^ was used to identify differentially expressed genes (DEGs) between FMT water extract and vehicle control groups with the following cutoffs values: *p* < 0.05, absolute fold change >2, and the mean channel intensity more than 250.

### Ingenuity Pathways Analysis and Real-Time Quantitative PCR (RT-qPCR)

The functions of differentially expressed mRNAs were analyzed using Ingenuity Pathway Analysis software (IPA, Ingenuity Systems, QIAGEN, Hilden, DE). The intersection of the predicted target genes of differentially expressed mRNAs, biological and molecular functions of these genes was identified by IPA. The resulting candidate genes were further examined by Real-time Quantitative PCR (RT-qPCR) from tissue and cell samples. RT-qPCR was carried out with the same RNA samples used for microarray analysis and 22 genes were selected to validate the results from the mRNA microarray using real-time quantitative PCR with an Eco™ Real-Time PCR System (Illumina, San Diego, CA, United States) and the specific primers were shown in Table 1. Results are expressed as mean of fold change compared to wild-type mice.

**TABLE 1:** List of qRT-PCR primers.

Gene symbol	Forward (F) and Reverse (R) Primers	Primer sequences
*mat1a*	F	GTG​ATG​CGG​TGC​TGG​ATG​CT
	R	GCC​AAT​GTG​CTT​GAT​GGT​GTC​T
*me1*	F	GCC​CTG​AGT​ATG​ACG​CCT​TCC​T
	R	AGA​CCC​GCA​ACC​GCA​ACA​GA
*acss2*	F	AGT​GTG​AGC​CTG​AGT​GGT​GTG​A
	R	ACC​AGT​GAT​CCA​GCC​GAT​GTC​T
*ass1*	F	CAA​AGC​ACC​CAA​CAG​CCC​AGA​T
	R	GAA​GCG​GTT​CTC​CAC​GAT​GTC​A
*Gnmt*	F	CGA​CCA​CCG​CAA​CTA​CGA​CTA​T
	R	ACT​GAA​GCC​AGG​AGA​GCC​ATC​T
*cps1*	F	TGG​CTT​AGG​CTC​TGG​CAT​CTG​T
	R	AAC​CTG​TCA​CTG​ACC​GCT​CCA
*pnpla3*	F	TGG​ACG​GAG​GAG​TGA​GCG​ACA​A
	R	GCG​GAG​GCT​GAG​GTT​GGT​GAT​A
*elovl2*	F	GCC​TGT​CTG​TGT​TCC​CGT​CCA​T
	R	AGC​GTG​TGC​GTG​ATG​GTG​AGT​A
*Lpl*	F	GAG​GAT​GGC​AAG​CAA​CAC​AAC​C
	R	AGC​AGT​TCT​CCG​ATG​TCC​ACC​T
*cyb5r3*	F	GGA​GGA​GCC​GCT​GAT​ACT​GAT​G
	R	GGA​CAG​GTG​GTT​GTG​GTG​AGT​G
*pik3ca*	F	ATG​ATG​CTT​GGC​TCT​GGA​ATG​C
	R	TGC​TGC​TTG​ATG​GTG​TGG​AAG​A
*Gapdh*	F	GAA​GGT​GGT​GAA​GCA​GGC​ATC​T
	R	CGG​CAT​CGA​AGG​TGG​AAG​AGT​G
*Myc*	F	TGT​GGT​GTC​TGT​GGA​GAA​GAG​G
	R	GGC​GTA​GTT​GTG​CTG​GTG​AGT
*fads1*	F	ACA​GTT​CAG​GCT​CAG​GCA​GGT​T
	R	TAG​GCT​TGG​CAT​GGT​GCT​GGA​A
*fads2*	F	TGC​CTT​CCG​TGC​CTT​CCA​TCT
	R	GCC​CTG​AAG​TCC​TCG​GTG​ATC​T
*Fasn*	F	CCA​GAG​GCT​TGT​GCT​GAC​TTC​C
	R	TTG​TGG​CTT​CGG​CGA​TGA​GAG
*Acacb*	F	CAG​CCA​GCA​GAT​AGC​CAC​CAT​C
	R	GTC​CAG​CAC​CAC​AGC​CTT​CAT
*fabp5*	F	GGA​CGG​GAA​GGA​GAG​CAC​GAT​A
	R	TGT​CCA​GGA​TGA​CGA​GGA​AGC​C
*tm7sf2*	F	TCG​CCT​CGG​TTC​CTT​TGA​CTT​C
	R	AGC​CAT​TGA​CCA​GCC​ACA​TAG​C
*Lepr*	F	CGG​AGA​GCC​ACG​CAA​CTT​CT
	R	CAA​ACC​AAC​CCA​CCC​TCT​TTC​C
*g6pdx*	F	AAG​CAC​CTC​AAC​AGC​CAC​ATG​A
	R	CTC​CAC​GAT​GAT​GCG​GTT​CCA
*lc15a4*	F	GCT​GCC​TCA​TGT​CTG​CTT​CTC​A
	R	TGC​GTG​GTG​TAA​CTG​CCA​ATC​T

### Statistics

The statistical tests used in each analysis are stated in the corresponding figure legends. Statistical analyses were performed using a *t*-test or one-way ANOVA depending on experimental groups. Error bar in all figures are represented as standard deviation (SD).

## Results

### KLF10 Deficiency Exacerbates Lipid Accumulation and NAFLD Pathogenesis

**FIGURE 1:**
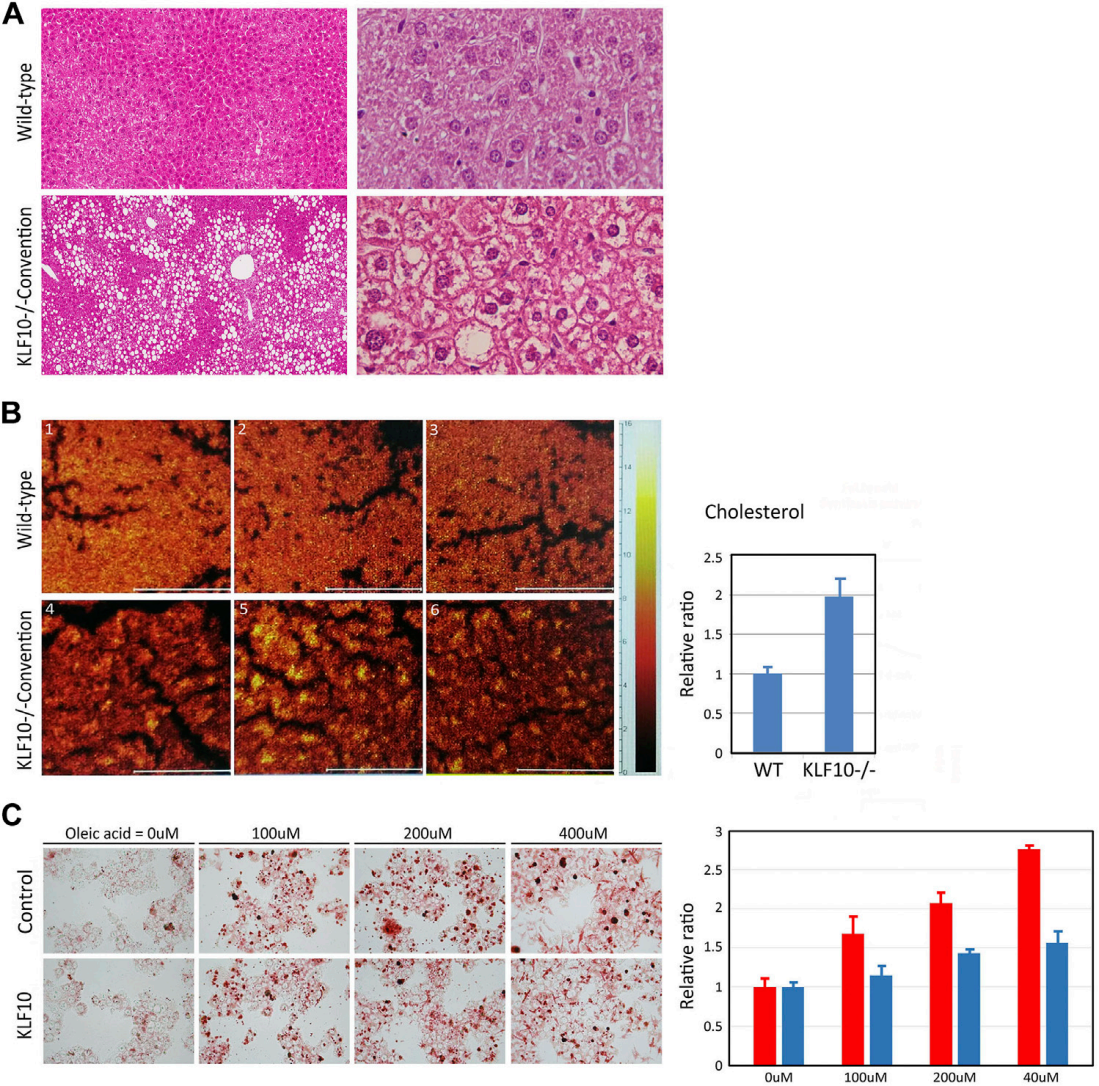
KLF10 deficiency causes lipid accumulation *in vivo* and *in vitro*. **(A)** Representative liver sections in control and KLF10-knockout mice treated with HFD (**left panels**, 100X; **right panels**, 400X) with H and E stain. **(B)** Optical micrographs with superimposed cholesterol mouse hepatic tissues. The images were overlaid to observe the colocalization of cholesterol. The right panel indicates area-normalized intensities of a cholesterol for standard samples and subareas in tissue sections. The results represented as mean ± SD (n = 3). **(C)** Lipid droplet formation was observed by oil-red-O staining in control and HepG2 cells transfected with KLF10 after treated with OA (0–400 mM). The quantification of triglyceride contents in cells treated with OA **(right panel)** in control **(red column)** and KLF10 overexpressed (blue column) HepG2 cells. Data represented as mean ± SD (n = 3).

KLF10 signaling is associated with metabolic regulation, which plays an important role in lipid and carbohydrate metabolism, glucose utilization and insulin resistance (Guillaumond et al., 2010; Pollak et al., 2018). Therefore, we first assessed the potential effects of KLF10 in a HFD model of NAFLD and their hepatic histology was then examined. As shown in Figure 1A, HFD resulted in slight hepatic lipid accumulation and led to hepatic steatosis in wild-type mice; however, liver damage was aggravated in KLF10-knockout mice, which exhibited an accumulation of lipid droplets and degeneration of ballooning in the liver. Increased lysosomal cholesterol storage inside hepatocytes is correlated with several metabolic disorders including NAFLD. (Hwang et al., 2013). Therefore, the liver cholesterol level was detected in KLF10-knockout and wild-type mice (Figure 1B). Furthermore, oleic acid (OA) which is known to induce morphological similarities to steatotic hepatocytes through lipid accumulation, were treated to HepG2 cells to develop a cellular model of hepatic steatosis. As shown in Figure 1C, compared with control cells, KLF10-transfected HepG2 cells showed significant less recovered oil red O content (determined by MOD) (Figure 1C) suggested that overexpression of KLF10 rescued steatosis in liver cells. The results indicated the regulatory effects of KLF10 on lipid accumulation *in vitro* and *in vivo*. Next, we explored the mechanisms underlying KLF10 signaling in NAFLD.

### KLF10 Is an AMPK Interacting Protein in Cells and Is Phosphorylated by AMPK

**FIGURE 2:**
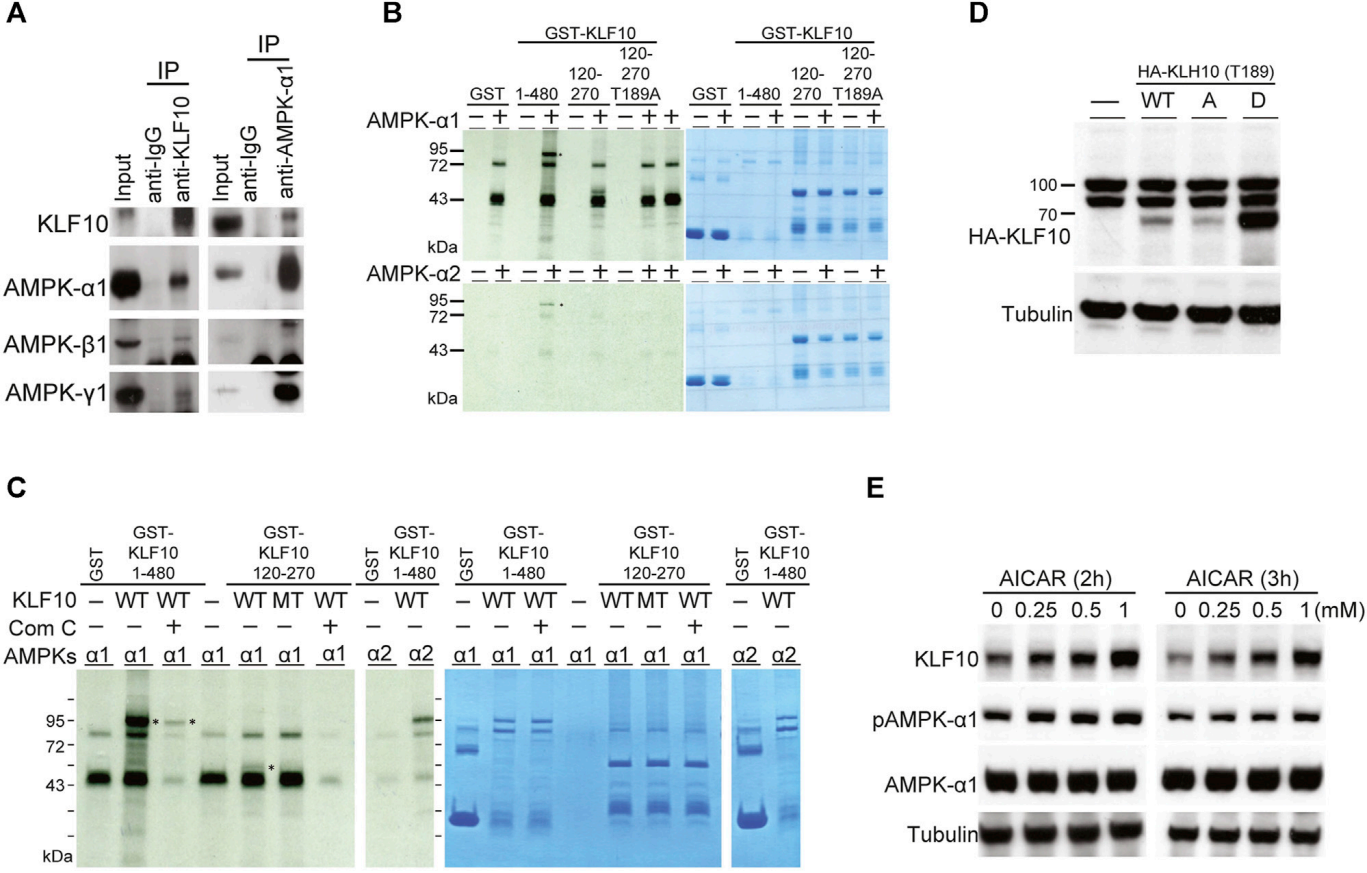
KLF10 associated with AMPKs in *in vivo* and *in vitro* studies. **(A)** Immunoprecipitation was performed with extracts of HepG2 cells using either anti(a)-KLF10 or normal mouse immunoglobulin as the negative control. Precipitates were identified using a-KLF10, and the association was detected NGA-AMPK α1, *β*1, and *γ*1. **(B)** The *in vitro* association of AMPK *α*1 and *α*2 with KLF10 was analyzed *via* GST retention. Flag-tagged AMPKs were individually transfected into HepG2 cells for 24 h. Proteins retained on Sepharose were then subjected to Western blotting by using an *a*-flag antibody. **(C)** HepG2 cells expressing GST–KLF10 with overexpression of AMPK *α*1 or *α*2 were treated compound C for 16 h. The cell lysates were then precipitated with anti-KLF10 and analyzed by Western blotting using anti-GST antibodies. **(D)** HepG2 cells were transiently transfected with pRK5-flag-KLF10 wild-type, Thr189A, or Thr189D for 24 h and then analyzed by Western blotting. **(E)** HepG2 cells pretreated with 0.25–1 μM AICAR for 2 and 3 h then the KLF10 levels were detected and quantified using tubulin as the loading control. The relative KLF10 levels were compared to the 0 h results.

To further evaluate the regulatory mechanisms of KLF10 activity, we identified that KLF10 may be phosphorylated by AMPK using a computational prediction program (PhosphoMotif; http://www.hprd.org/PhosphoMotif_finder). AMPK is an established heterotrimeric complex comprising a catalytic AMPKα subunit and regulatory AMPKβ and AMPKγ subunits. Frist, we investigated the physical association between KLF10 and AMPK by Co-IP assays using specific antibodies that can detect AMPK subunits. The results revealed that KLF10 and AMPK could form a complex indicated a direct interaction between them (Figure 2A). The various phosphorylation sites of both endogenously and ectopically expressed KLF10 was found in our previous studies (Hwang et al., 2013). However, the exact phosphorylation site of KLF10 by AMPK has never been studied. We further examine whether AMPK catalytic subunits could phosphorylate KLF10, an *in vitro* kinase assay was performed using either GST alone or the GST–KLF10 fusion protein as the substrate and active recombinant AMPK subunits (α1 and α2) as the kinase. The reaction was conducted in the presence of radiolabeled [γ-33P] ATP as a phosphate donor. Incubating the recombinant GST–KLF10 protein with AMPK α1 and α2 resulted in a phosphorylated GST–KLF10 band (1–480 amino acids; Figure 2C, lane 4 in upper and lower panels, indicated as asterisk mark). This band was absent when neither AMPK α1 nor α2 was not added (lanes 3, 5, and 7 in both panels, Figure 2B) or when the GST–KLF10 protein was incubated with a Thr189 mutant (mimic dephosphorylated status, named as Thr189A, lanes 8 and 9, Figure 2B). To further clarify the role of AMPK in KLF10 phosphorylation, AMPK inhibitor compound C was used and significantly reduced the levels of phosphorylated KLF10 (lane 3, indicated as asterisk mark) confirmed that AMPK is a kinase for KLF10 phosphorylation (Figure 2C).

We next test whether AMPK and KLF10 interaction has any biological significance. HepG2 cells were co-transfected with wild-type KLF10, Thr189A (mimic dephosphorylated status; A: alanine acid) or Thr189D (mimic phosphorylated status; D: aspartic acid) mutant, and subsequently detected KLF10 expression levels (Figure 2D). Compared with the phosphor-defective mutant Thr189A, the phosphor-mimetic Thr189D increased KLF10 expression (Figure 2D). Similar effects were detected in cells when AMPK agonist AICAR treatment for 2 or 3 h induced AMPK α1 activation and increased the expression of KLF10 in a dose dependent manner (Figure 2E). Taken together, the results revealed that AMPK has a phosphorylation-dependent association with KLF10 and confirm the essential role of Thr189 phosphorylation in regulating KLF10 stability and expression.

### KLF10 Downregulates the Levels of SREBP-1C *via* AMPK Phosphorylation

**FIGURE 3:**
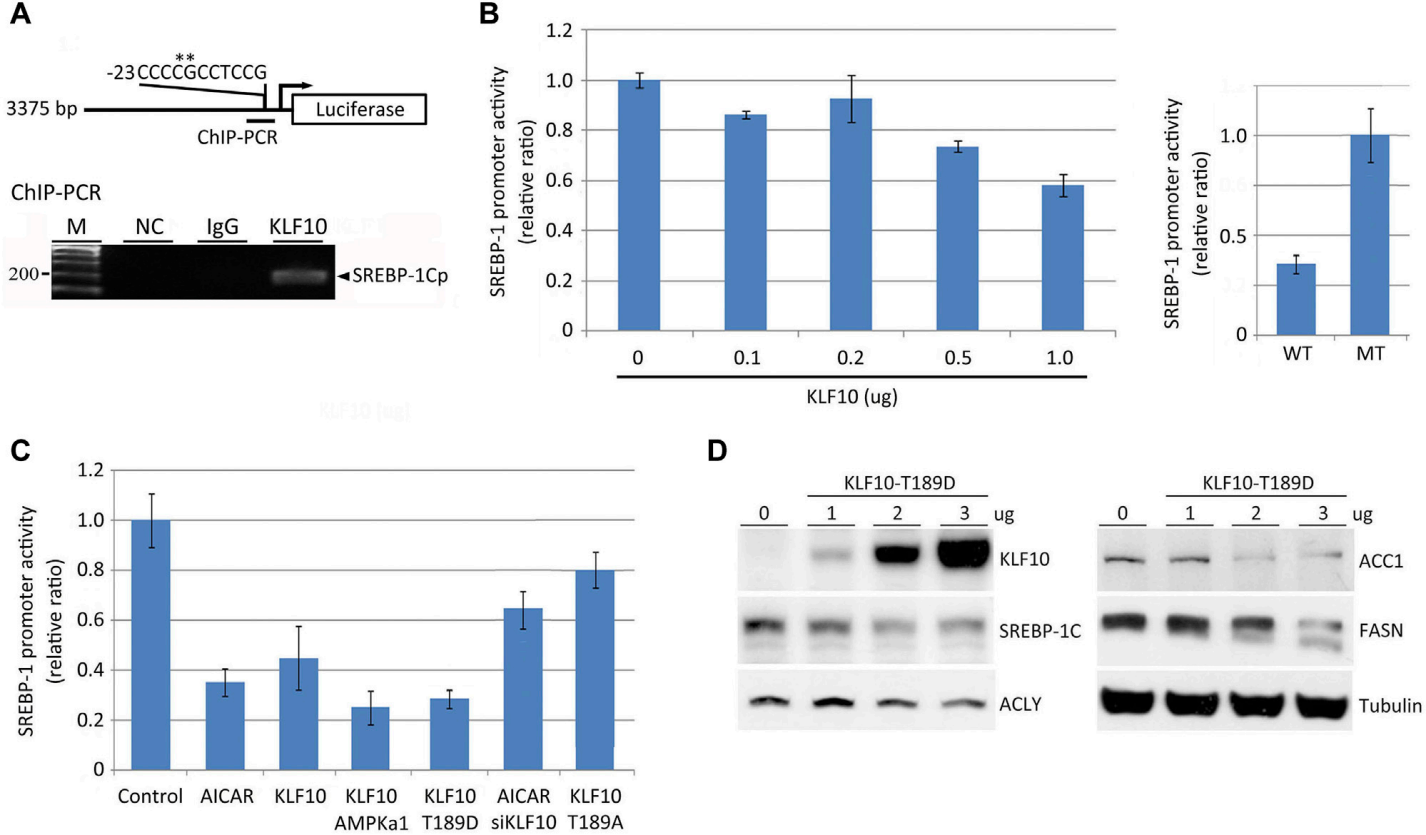
KLF10 modulates SREBP-1C promoter activity *via* direct binding. **(A)** A diagram of the cloned 3.3-kb human SREBP-1C promoter showing the SP/KLF binding site from positions−13 to−23. An expression plasmid encoding KLF10 and the SREBP-1 promoter luciferase reporter, the latter with (MT) or without (WT) site-directed mutagenesis, was transfected into HepG2 cells to conduct transcriptional reporter assays. **(B)** HepG2 cells were individually transfected with a wild-type SREBP-1C promoter–luciferase construct with or without a KLF10-overexpressing construct (0.1–1.0 μg) for 12 h. Cellular extracts were used to measure luciferase activity and normalized against Renilla luciferase activity. Data are represented as the mean ± SD (n ≥ 3). In the right panel, wild-type or site-directed mutant constructs of the SREBP-1C promoter were transfected into HepG2 cells co-transfected with KLF10 (1.0 μg) and the luciferase activity was detected. **(C)** The quantification of transcript levels of SREBP-1C after HepG2 cells treated with AICAR or transfected with KLF10, AMPK, mutant KLF10 constructs (T189D and T189A), or siKLF10. **(D)** The expression of KLF10, SREBP-1C, ACLY, ACC1, and FASN were detected after transfected with KLF10-189D mutant for 48 h. Tubulin was used as a loading control.

A ChIP–chip assay was used to screen for possible targets of KLF10 based on its specific binding sites for transcription regulation (Hsu et al., 2011). Approximately 1,200 genes showed altered binding interactions when KLF10 was ectopically expressed (Hsu et al., 2011). We observed that KLF10 may regulate SREBP-1C by binding to its promoter. In this context, an SP/KLF binding site was predicted in the SREBP-1C promoter region (located at−13 to−23) by using TFsearch (http://www.cbrc.jp) (Figure 3A). Through ChIP–PCR, a cell extract from KLF10 overexpressing cells was retained by KLF10 antibody and then subjected to PCR, which revealed a clear 200-bp product (Figure 3A, lower panel). This PCR fragment was further sequenced and aligned to the SP/KLF region of SREBP-1C. To elucidate the specificity of the KLF10 binding site on the SREBP-1C promoter and to evaluated whether KLF10 reduces the expression levels of SREBP-1C, HepG2 cells were transfected with a deletion mutant construct (Figure 3A) of the SREBP-1C promoter which was created by inducing point mutations at −20 and −19 from GC to AA (labeled with an asterisk, Figure 3A). As expected, KLF10 at higher doses significantly reduced SREBP-1C promoter activity; however, SREBP-1C containing the mutation at the SP/KLF site remained promoter activity indicated KLF10 binds to SREBP-1C promoter then suppressed its activity (Figure 3B).

We next determine whether AMPK activation suppresses SREBP-1C mRNA levels through KLF10. As indicated in Figure 3C, AICAR induced activation of AMPK and subsequently reduced SREBP-1C transcripts by 60% in cells. Cells transfected with AMPK α1 or KLF10 Thr189D also reduced SREBP-1C promoter activity (column 4 and 5, Figure 3C). In contrast, AMPK could not inhibit SREBP-1C transcripts when KLF10 was silenced even in the presence of AICAR (column 6). A similar effect was observed in cells bearing KLF10 Thr189A (column 7; phosphor-defective status). Moreover, the translational level of SREBP-1C and its regulated key lipogenic enzymes including ATP citrate lyase (ACLY), ACC1, and FASN was decreased in KLF10 Thr189D transfected cells (Figure 3D). The results provide strong evidence that AMPK-KLF10 axis repressed the expression of SREBP-1C by binding to its promoter and subsequently downregulated lipogenesis, whereas KLF10 deficiency enhanced NAFLD.

### KLF10 Deficiency Alters the Expression of Hepatic Genes Involved in Lipid Metabolism

**FIGURE 4:**
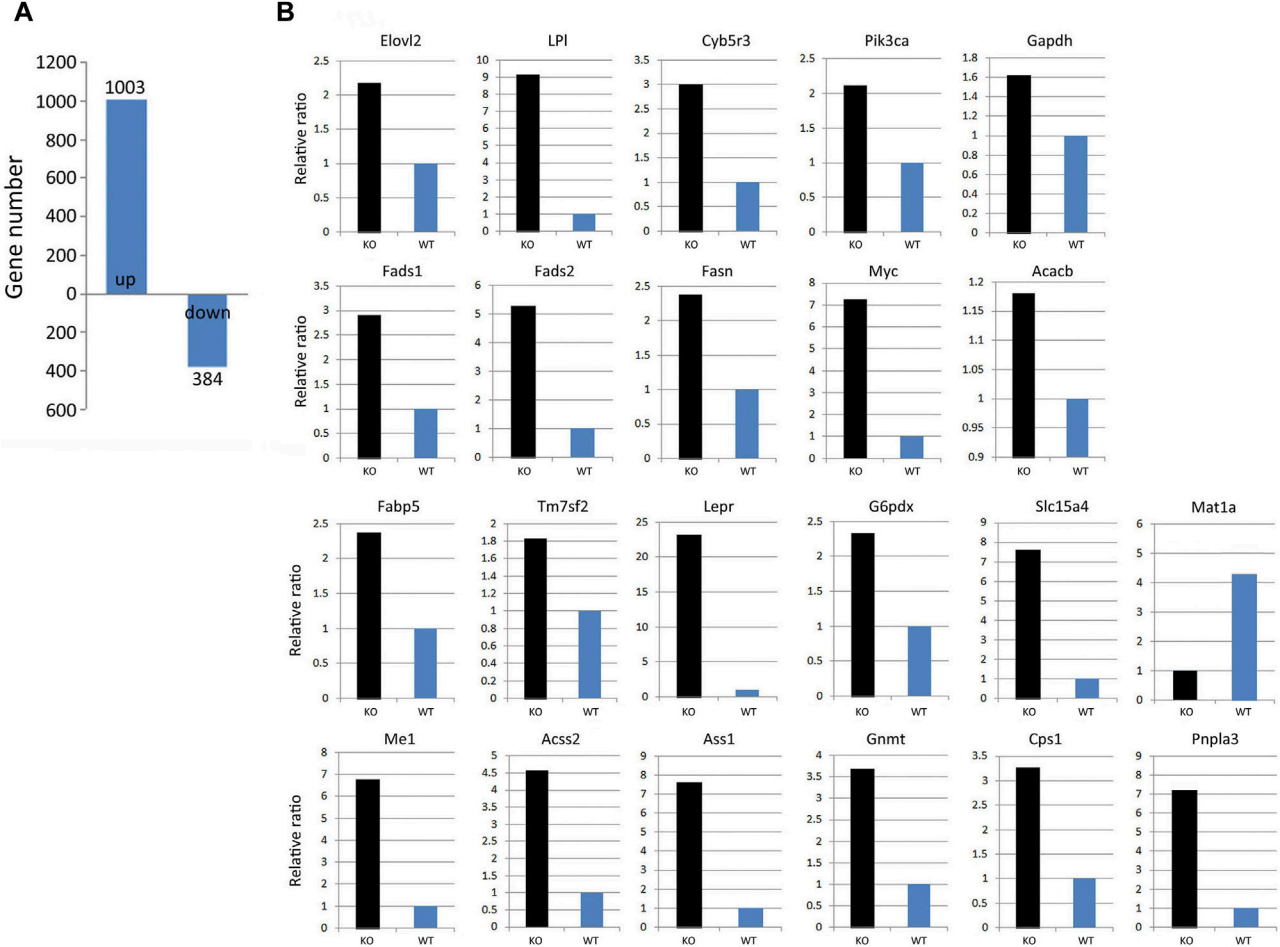
KLF10 deficiency alters gene regulation in the liver. **(A)** Number of genes with upregulated or downregulated expression in KLF10-knockout mice compared with wild-type mice (*n* = 3). **(B)** Array validation was performed with qPCR for 22 selected genes. The upregulated *Pnpla3* gene and downregulated *Mat1a* gene was used as NAFLD makers.

To understand the sequential mechanism by which KLF10 deficiency causes lipid accumulation, the hepatic gene expression pattern associated with KLF10 deficiency in mice fed a HFD were detected. According to microarray data, 1,003 upregulated and 384 downregulated genes (false discovery rate <0.05) were found in KLF10-knockdown mice (Figure 4A). Functional categorization and clustering the changed genes upon KLF10 deficiency were assigned according to Ingenuity Pathway Analysis, which revealed lipid metabolism as the leading network. As shown in Figure 4B, we validated 20 selected transcripts through qRT-PCR, which revealed a high correlation between the fold-change values and microarray data. The levels of markers of NAFLD were either upregulated (*Pnpla3*) or downregulated (*Mat1a*) (Figure 4B). The results further showed that KLF10 deficiency significantly increased the levels of the known SREBP-1 lipogenic target genes *Acacb* (approximately 1.2-fold), *Fasn* (approximately 2.5-fold), and markedly enhanced the expression levels of metabolic genes. These results suggested that KLF10 played a vital role in the regulation of genes associated with lipid metabolism and NAFLD.

**FIGURE 5:**
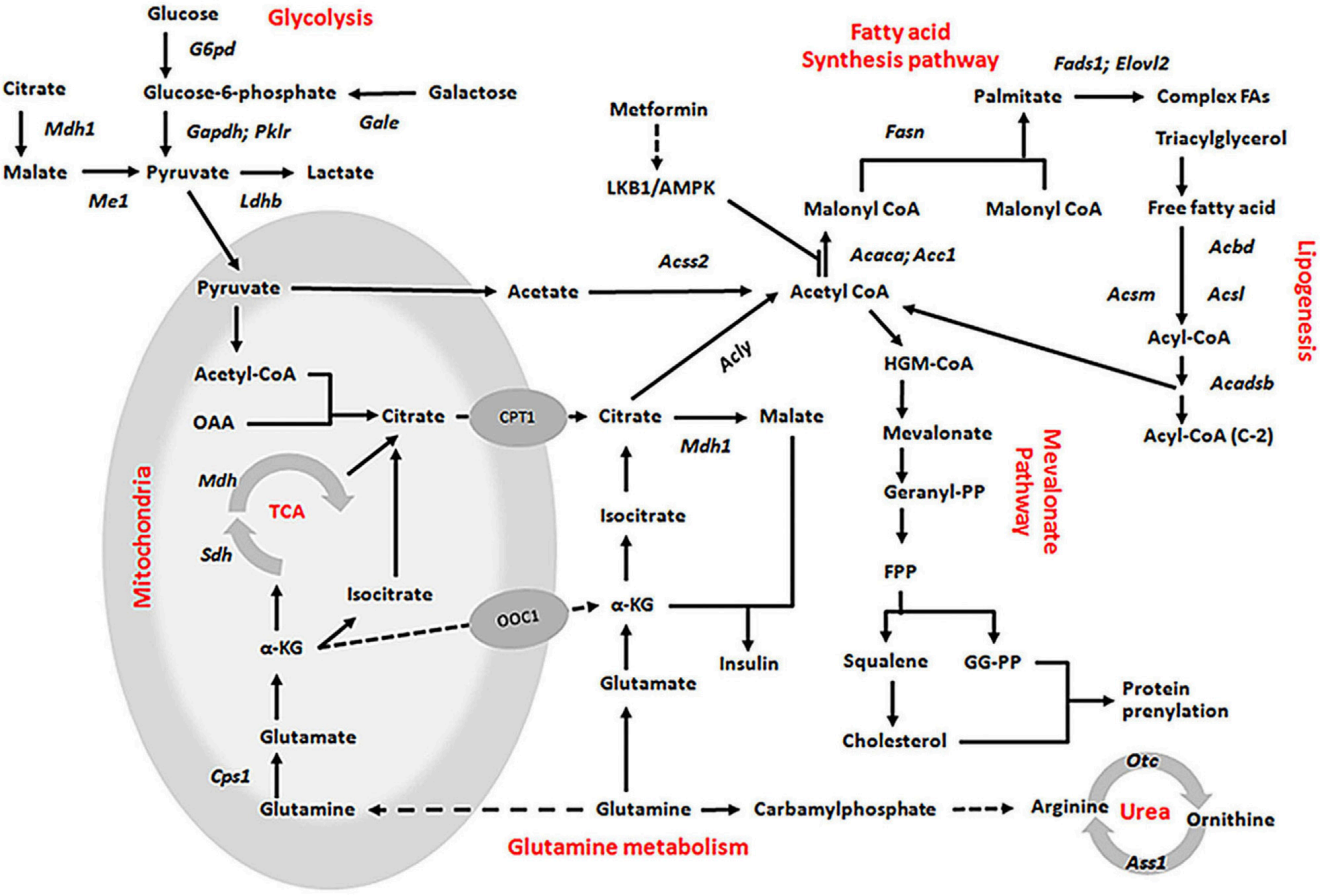
Proposed mechanisms for the role of KLF10 in the lipogenesis, glycolysis, and glutamine metabolism pathways. All indicated genes with italic words were upregulated by KLF10, which presented a significant fivefold change in the expression array.

**FIGURE 6:**
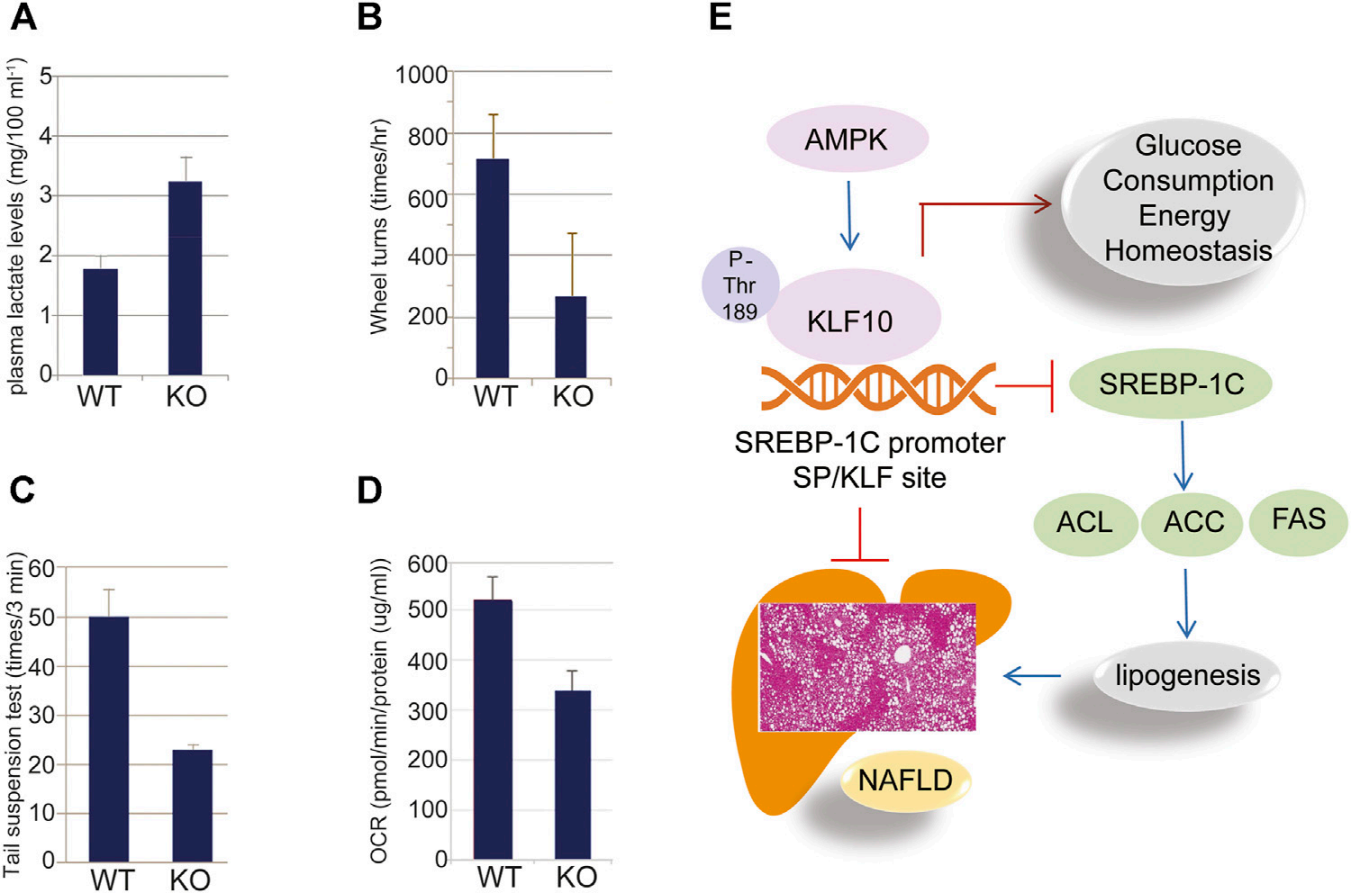
KLF10 regulated glucose metabolism in mice. **(A)** Plasma lactate levels in wild-type (WT) and KLF10^−/−^ (KO) mice. **(B)** KLF10^−^KO mice traveled a significantly shorter distance than WT mice that was detected by wheel turns experiment. **(C)** KLF10-deficient mice exhibited shorter immobility times in the tail suspension test. **(D)** Seahorse X-24 analysis of the oxygen consumption rate (OCR) in WT and KLF10-KO mouse livers under basal conditions. All data are represented as mean ± SD. (*n* = 6) **(E)** The proposed scheme of NAFLD prevented by AMPK-KLF10 axis. AMPK phosphorylates KLF10 at Thr189 leading to enhanced stability and activity. Activated KLF10 inhibits SREBP-1C expression by binging to its promoter thereafter reduced the expression of lipogenesis related genes including ACL, ACC, and FAS, ultimately contributed to the prevention of NAFLD.

In addition, we found that glycolytic pathways may also affected by KLF10. According to our studies, KLF10 deficiency increased the mRNA expression levels of several glycolytic and other lipogenetic markers in mice fed with HFD (Figure 5; italicized words). The scheme also shows central carbon metabolism changes through enzymatic alteration resulted in an anaerobic track in KLF10-knockout mice. Substantial evidence indicates increased glucose consumption and lactate secretion in this scheme that lactate could be a marker for distinguishing metabolic disorders *in vivo*. The results showed that plasma lactate levels were higher in KLF10-knockout mice compared with control groups measured after exercise, indicating increased carbohydrate consumption in KLF10-knockout mice (Figure 6A). Meanwhile, we observed that knockout mice were exercise intolerant with markedly decreased endurance to exhaustion (Figures 6B,C). We then examined mitochondrial function in the livers of mice and found that the oxygen consumption rate (OCR) was decreased in the absence of KLF10 (Figure 6D). In summary, the results suggested that KLF10 modulates lipogenesis through AMPK–SREBP-1C signaling pathways, energy homeostasis and consumption, while deficiency of KLF10 leads to NAFLD and impairment of energy homeostasis.

## Discussion

The prevalence of NAFLD increasing worldwide that ranging from 13% in Africa to 42% in Southeast Asia (Huang et al., 2021). NAFLD encompasses a spectrum of diseases including simple steatosis or nonalcoholic fatty liver (NAFL), the nonalcoholic steatohepatitis (NASH) which is a more progressive and severe stage of NAFLD and is often accompanied by fibrosis that can progress to cirrhosis or hepatocellular carcinoma (HCC) (Huang et al., 2021). The development of NAFLD and the following NASH is a complex process that accumulation of lipid droplets within hepatocytes occurs as a result of a dysregulated lipid metabolism, which is closely associated with a metabolic syndrome including obesity, insulin resistance, dyslipidemia and hypertension (Filali-Mouncef et al., 2021). So far, the molecular basis of this disease entity is still unclear and the effective preventive or therapeutic agents for NAFLD is still lacking. Among the signaling pathways, AMPK is well established as an important mediator of energy metabolism that is involved in the development of NAFLD (Chyau et al., 2020). Understanding the complex regulatory mechanisms of AMPK-regulated pathways may increase the opportunity to develop better effective drugs to prevent NAFLD, thereafter decreasing the incidence of further liver diseases such as NASH, fibrosis, and cancer. In the present study, we demonstrated that liver damage, such as lipid accumulation and steatosis, was aggravated in KLF10-knockout mice (Figure 1). We confirmed the results by using KLF10 transfected HepG2 cells and showed that compared to the control cells, KLF10 overexpressed cells significantly reduced lipid accumulation and steatosis. The results strongly implicated the protective role of KLF10 in the development of NAFLD. The present work demonstrated for the first time that KLF10 may act as a novel substrate for AMPK in regulating hepatic lipogenesis through a posttranslational modification and act as a transcriptional repressor of SREBP-1C. In addition to SREBP-1C pathways, we also suggested KLF10 could regulate various signaling relevant to lipid and glucose metabolism (Figure 6E). Therefore, we highly suggested that KLF10 is a key regulator of the metabolism and also could act as a therapeutic target for intervention on NAFLD.

AMPK is well established as an important mediator of energy metabolism that is involved in regulating several pathways related to NAFLD (Chyau et al., 2020). For instance, the phosphorylation of the SREBP-1 precursor at Ser372 by AMPK inhibits its nuclear translocation and transcriptional activity, resulting in the downregulation of fatty acid synthase (FAS) expression. In addition to the direct regulatory mechanisms of AMPK and SREBP-1C, several downstream mediators of AMPK were reported to suppress the expression of SREBP-1C, leading to inhibited lipid synthesis and insulin resistance (Chyau et al., 2020; Han et al., 2019). In this context, we clearly demonstrated that AMPK phosphorylated KLF10 at Thr189 thereafter induced a protein interaction with AMPK (Figure 2). Phosphorylation of KLF10 following AMPK activation was abrogated by the inactivating mutant Thr189A, indicating that Thr189 is specifically phosphorylated in an AMPK-dependent manner (Figure 2). In addition, the expression of KLF10 in HepG2 cells transfected with KLF10 was increased in response to treatment with AMPK agonist AICAR (Figure 2), confirming that the Thr189 site is essential for the stabilization of KLF10. Previous studies have also indicated that the function of KLF10 is highly regulated by its phosphorylation and ubiquitination. For instance, we have reported that KLF10 was phosphorylated by CDK2 and Raf-1 at Ser206 and Thr93, respectively, further affecting the role of KLF10 in tumor suppression (Hwang et al., 2013; Lin et al., 2015). Another study indicated that KLF10 was degraded by the E3 ubiquitin ligase FBW7 after phosphorylation at Thr82-87 (Yu et al., 2018). The results confirmed that phosphorylation of KLF10 affected its expression and activity, while AMPK definitely is an upstream regulator for the KLF10.

Several studies have indicated that AMPK-ACC, AMPK-mTOR, AMPK-PPARγ, and AMPK-SIRT1 pathways are important for the regulation of SREBP-1C (Liu et al., 2019; Song et al., 2019; Teng et al., 2019). AMPK and mTOR inhibited transcriptional activation of SREBP-1C *via* phosphorylation (Moslehi and Hamidi-Zad, 2018; Zhu et al., 2020). In this context, natural products such as dietary flavonoids could suppress SREBP-1C expression and the following enzymes involved in lipid synthesis thereby provided benefits in the prevention of NAFLD (Moslehi and Hamidi-Zad, 2018). However, despite SREBP-1C has emerged as one of the therapeutic targets for NAFLD, the role of its transcriptional regulation is still largely unknown. Herein, we have revealed a novel mechanism of KLF10 in the transcriptional repression on SREBP-1C. Indeed, we showed for the first time that KLF10 reduces SREBP-1C expression by binding to the SP/KLF site on the SREBP-1C promoter (Figure 3). A dose-dependent decrease in SREBP-1C expression was detected in phosphorylated KLF10-transfected cells (Figure 3). Afterward, the expression of genes downstream of SREBP-1C and related to lipogenesis, such as ACL, ACC, and FAS, was inhibited. In contrast, AMPK activation by an AMPK activator had no direct effects on SREBP-1C transcripts when KLF10 was silenced (Figure 3). These solid evidences indicated that KLF10 acts as a transcriptional repressor of the expression of SREBP-1C, whereas KLF10 depletion is sufficient to induce NAFLD. Moreover, the phosphorylation of Thr189 on KLF10 by AMPK is necessary to increase the stability and the transcriptional repressor activity of KLF10 leading to reduced SREBP-1C expression.

Herein, we suggest that AMPK-KLF10 axis regulated SREBP-1C expression and the following lipid metabolism gene expression. It was report that SREBP-1C regulates the expression of *Pnpla3*, leading to increased lipid accumulation and disrupted with lipolysis (BasuRay et al., 2017). Consistently, our results showed that *Pnpla3* was increased in the KLF10 deficient groups in which the expression of SREBP-1C was increased (Figure 4). In addition, the expression of the SREBP-1C target genes *Acacb* and *Fasn,* which are involve in FA binding and oxidation (Seo et al., 2020; Turner et al., 2020; Yang et al., 2020), also show upregulated in response to downregulation of KLF10 expression (Figure 5). These findings were consistent with previous findings showing that KLF10 expression was induced after treatment with a PPARα agonist, which promotes FA oxidation in primary mouse hepatocytes (Rakhshandehroo and Hooiveld, 2009). Although the direct interaction between PPARα and KLF10 has not been established, we could provide a potential role showing that KLF10 activation regulated genes involved in FA oxidation. Moreover, KLF10-silenced groups exhibited overexpression of *Cyb5r3*, *Fads1*, and *Fads2*, which are involved in diseases such as lipid metabolism disorder (Jakobs et al., 2014; Koletzko et al., 2019). Therefore, we could provide a potential role showing that KLF10 activation regulated genes involved in lipogenesis and FA oxidation. Taken together, the results suggest that KLF10 downregulated SREBP-1C through phosphorylation by AMPK, thereby regulating the complicated lipogenesis pathways to prevent the development of NAFLD.

There is a growing concern with glucose metabolism, insulin resistance and their association with NAFLD (Guillaumond et al., 2010). As described in previous studies, KLF10 also acts as an inhibitor of gluconeogenesis by directly inhibiting the *Pepck* gene. Accordingly, KLF10 deficient mice showed an elevation in blood glucose and TG levels (Guillaumond et al., 2010). Meanwhile, the expression of genes regulating energy homeostasis and glucose metabolism, including *Lepr* and *Acss2*, was found to be increased in KLF10-deficient groups (Moffett et al., 2020). Consistently, we demonstrated that KLF10 deficient mice were exercise intolerant and decreased endurance to exhaustion resulted from alteration of the genes associated with carbohydrate metabolism including *G6pd, Ldhb*, Mdh, *Sdh*, and *Cps1* genes (Figures 5, 6). These results could partially explain the findings that KLF10-deficient mice exhibited lower energy homeostasis and less endurance to exhaustion (Figure 6). Moreover, AMPK and its regulated genes including SREBP and ChREBP were involved in the regulation of metabolic disorders such as hyperglycemia, hypercholesterolemia, hyeruricemia that is associated with kidney diseases, cardiovascular disease, and NAFLD. In addition, Iizuka et al. indicated that the crosstalk between ChREBP and KLF10 is involved in the regulation of the lipogenic pathway (Iizuka et al., 2011). Accordingly, we suggest that KLF10 could be a critical target for resolving these metabolic disorders. In addition to the role in the regulation of metabolism, KLF10 also acts as a tumor suppressor through the TGF-β/Smad signaling pathway by inhibiting cell proliferation and inducing apoptosis (Memon and Lee, 2018). Collectively, previous findings along with ours highlighted that KLF10 plays an active role in the regulation of various diseases and appears to have unique tissue-specific roles. In summary, the current study identified a biochemical mechanism of KLF10 regulation by AMPK. AMPK activates and stabilizes the KLF10 protein *via* phosphorylation at Thr189, thereby repressing the expression of SREBP-1C and subsequent lipogenesis pathways and metabolic disorders. Impairment of KLF10 function is associated with lipid accumulation and alterations of glucose metabolism, suggesting that activation of the AMPK-KLF10 axis could be a potential therapeutic target for the treatment of metabolic syndrome, including NAFLD. In contrast to our findings, a previous study indicated that KLF10 expression was significantly increased in diet-induced NASH accompanied by increased TGF-β signaling and suppressed ChREBP expression (Kim et al., 2014), however, the related mechanisms are still unclear. Therefore, further studies are needed to identify the consequences of abnormalities in the expression, structure, and/or function of KLF10 in NAFLD.

## Data Availability

The data presented in the study are deposited in Figshare, https://doi.org/10.6084/m9.figshare.16680409.v1.
